# A joint matrix minimization approach for seismic wavefield recovery

**DOI:** 10.1038/s41598-018-20556-1

**Published:** 2018-02-01

**Authors:** Liping Wang, Yanfei Wang

**Affiliations:** 1 Department of Mathematics, Nanjing University of Aeronautics and Astronautics, Nanjing, 210016 P. R. China; 2 Key Laboratory of Petroleum Resources Research, Institute of Geology and Geophysics, Chinese Academy of Sciences, Beijing, 100029 P. R. China; 3 University of the Chinese Academy of Sciences, Beijing, 100049 P. R. China

## Abstract

Reconstruction of the seismic wavefield from sub-sampled data is important and necessary in seismic image processing; this is partly due to limitations of the observations which usually yield incomplete data. To make the best of the observed seismic signals, we propose a joint matrix minimization model to recover the seismic wavefield. Employing matrix instead of vector as weight variable can express all the sub-sampled traces simultaneously. This scheme utilizes the collective representation rather than an individual one to recover a given set of sub-samples. The matrix model takes the interrelation of the multiple observations into account to facilitate recovery, for example, the similarity of the same seismic trace and distinctions of different ones. Hence an *l*_2, *p*_(0 < *p* ≤ 1)-regularized joint matrix minimization is formulated which has some computational challenges especially when *p* is in (0, 1). For solving the involved matrix optimization problem, a unified algorithm is developed and the convergence analysis is accordingly demonstrated for a range of parameters. Numerical experiments on synthetic and field data examples exhibit the efficient performance of the joint technique. Both reconstruction accuracy and computational cost indicate that the new strategy achieves good performance in seismic wavefield recovery and has potential for practical applications.

## Introduction

Reconstruction of the seismic wavefield has recently attracted increasing attentions in geophysical community. This is due to the fact that seismic acquisition often violates the Shannon sampling theorem because of the restrictions of investment, topography, noise, bad traces and so on. The under-sampled data will bring aliasing and artifacts which will influence results of migration^1^, de-noising^2^, multiple elimination^3^ and AVO analysis^4^. In addition, huge storage of the massive data is also a problem, lossless compression methods are desirable^5^. An important branch of these methods is the sparse transform based method combined with a regularization strategy^6,7^. For this method, seismic interpolation is treated as an inverse problem, and seismic events are assumed to be sparse in some transformed domain, such as the Fourier transform^1,8–11^, or the linear Radon transform^12^. Usually the acquired geophysical data is subsampled due to the variations of landform^1,13,14^, hence the seismic wavefield recovery is an ill-posed inverse problem. Therefore, a key issue is how to invert the mathematical model using only incomplete, sub-sampled data^1,13,14^. Variety of regularization methods has been developed to improve the quality of image and seismic wavefield recovery^6,15–17^.

Previous methods for such a recovery problem are based on the *l*_*q*_-norm minimization, e.g., the basis pursuit denoising (BPDN) criterion using (orthogonal) matching pursuit method^18,19^ and the least absolute shrinkage and selection operator (LASSO)^20^ for *l*_1_-norm constrained minimization problems. Efficient optimization algorithms include conjugate gradient methods with preconditioning techniques^21^ and gradient projection methods^22–26^. For solving the *l*_*q*_-norm minimization problem, people usually convert the matrix form of the wavefield into the vector form and solve the corresponding matrix-vector equations. We observed that the seismic wavefield can be represented using matrix instead of vector as weight variable to express all the signals simultaneously, which takes the interrelation of the sampled observations into account. This is more reasonable as the seismic signals are correlated transversely. Therefore, in this paper we propose a matrix optimization model for the seismic wavefield recovery and study the related properties. The mixed matrix minimization models have been used in machine learning. Rakotomamonjy *et al*.^27^ proposed to use the mixed matrix norm *l*_*q*, *p*_ (1 ≤ *q* < 2, 0 < *p* ≤ 1) in multi-kernel and multi-task learning. But the induced optimization problems in^27^ have to be solved separately by different algorithms with respect to *p* = 1 and 0 < *p* < 1. For grouped feature selection, Suvrit^28^ addressed a fast projection technique onto *l*_1, *p*_-norm balls particularly for *p* = 2, ∞. But the derived method in^28^ does not match the proposed matrix optimization problem (11). Similar joint sparse representation has been used for robust multi-modal biometrics recognition in^29^. Sumit *et al*.^29^ employed the traditional alternating direction method of multipliers to solve the involved optimization problem. Wang *et al*.^30^ applied *l*_2, 0 +_ -norm to semi-supervised robust dictionary learning, while the optimization algorithm has not displayed definite convergence analysis^30^.

Recently, matrix-minimization methods with nuclear norm have been developed for seismic wavefield recovery^31–34^ which mainly considers the rank reduction as the sparse pattern in 2D cases. To avoid the expensive computations in solving the involved matrix completion optimization problems, a matrix factorization strategy was developed in^31,32^. This paper proposes a different matrix minimization approach based on *l*_2, *q*_−*l*_2, *p*_ norm which naturally generalizes the representative vector to matrix in joint distribution sense. A unified method is developed to solve the matrix optimization problem with mixed norm for any *q* = 2 and 0 < *p* ≤ 1. The innovations of this paper can be listed as follows:

1. A jointly sparse matrix minimization model is developed for seismic wavefield recovery. This approach employs matrix to expresses multiple signals simultaneously. The measurement of matrix row coefficients are expected to exhibit the compact priori of multiple observations which is different from the existed methods based on matrix nuclear-norm minimization^31–34^.
2. A unified algorithm is developed to solve the mixed matrix optimization problem (7) for any *p* $\in$ (0, 1]. This algorithm needs only matrix-vector operations but not matrix factorization which can be easily adapted to large-scale cases. The convergence analysis is also demonstrated.
3. Numerical experiments on synthetic and field data are carried out. The results on seismic wavefield recovery exhibit the efficient recovery performance of the joint sparse expression strategy.

## Modeling

Given a set of seismic signals (traces) *x*_1_, *x*_2_, …, *x*_*l*_ in *n*-dimensional space, each signal *x*_*j*_(*j* = 1, 2, …, *l*) is sensed by *m* sensors to yield seismic wavefield records as

1 $${d}_{ij}={A}^{i}{x}_{j},i=1,2,\cdots ,m,$$

where *A*^*i*^ is a row vector representing the impulse response of the *i*-th sensor. Denote *A* = [(*A*^1^)^*T*^, (*A*^2^)^*T*^, …, (*A*^*m*^)^*T*^]^*T*^, then the seismic observations *d*_*j*_ = [*d*_1*j*_, *d*_2*j*_, …, *d*_*mj*_]^*T*^ $\in$ *R*^*m*^ can be reformulated as *d*_*j*_ = *Ax*_*j*_(*j* = 1, 2, …, *l*). Sparse expression is a popular strategy to restore *x*_*j*_ with *m* much less than *n* of the mapping operator *A*.

**Figure 1:**
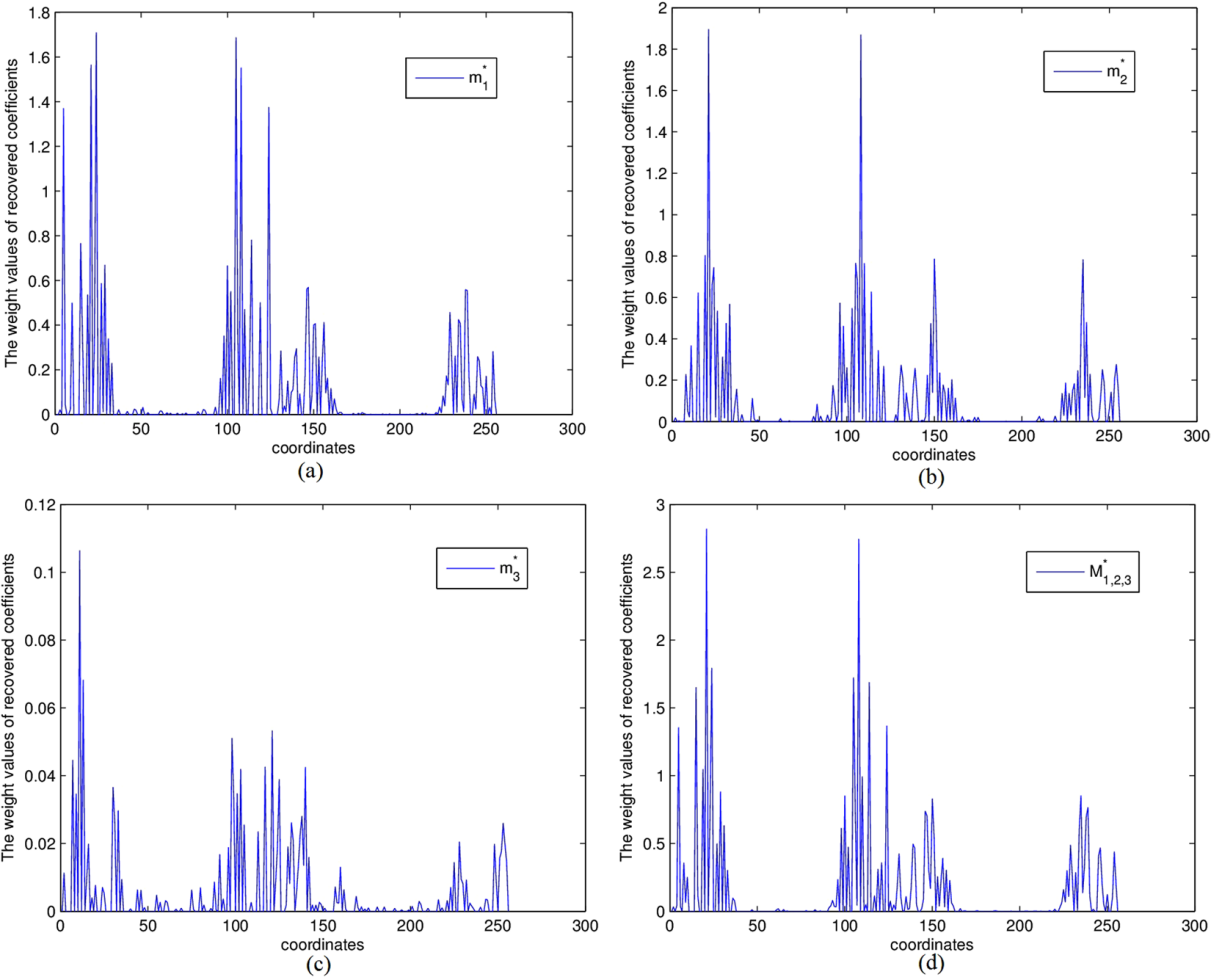
(**a**–**d**) denote the weight values of recovered coefficients of different traces.

Suppose that the original seismic signal *x*_*j*_ can be spanned by a series of orthogonal bases ${\{{{\rm{\Psi }}}_{k}\}}_{k=1}^{K}$ such that

2 $${x}_{j}(t)=\sum _{k=1}^{K}{m}_{j}^{k}{{\rm{\Psi }}}_{k},$$

where ${m}_{j}^{k}=({x}_{j},{{\rm{\Psi }}}_{k})$. Denote Ψ the orthogonal matrix constituted by the orthogonal bases, then we have a more compact transformation *L* = *A*Ψ $\in$ *R*^*m* × *K*^. Consequently the systems (1) and (2) can be incorporated to

3 $$L{m}_{j}={d}_{j},\,\,\,\,j=1,2,\cdots ,l,$$

where ${m}_{j}={\rm{\Psi }}\ast {x}_{j}={\{{m}_{j}^{k}\}}_{k=1}^{K}$ is the coefficient vector (weighting factor) corresponding to the seismic signal *x*_*j*_. Usually, problem (3) is ill-posed due to the limitation of acquisition and violation of sampling requirements. Sparse regularization is preferred to restore the operation coefficients from the under-determined linear combination system (3). A general *l*_*q*_−*l*_*p*_(*q* > 0, *p* > 0) model was presented in [16]

4 $$\mathop{\min }\limits_{{m}_{j}}\,{J}_{j}^{(\alpha )}({m}_{j})=\parallel L{m}_{j}-{d}_{j}{\parallel }_{q}^{q}+{\alpha }_{j}\parallel {m}_{j}{\parallel }_{p}^{p},\,\,\,\,q > 0,p > 0,$$

where $\parallel {m}_{j}{\parallel }_{p}^{p}=\sum _{k=1}^{K}|{m}_{j}^{k}{|}^{p}$ is the stabilizer bearing prior information with respect to *d*_*j*_ and *α*_*j*_ > 0 is a regularization parameter. When 0 < *p* ≤ 1, the minimization model (4) tries to find a sparse recovery coefficient *m*_*j*_ with the least nonzero entries. However, the framework (4) recovers the weight factor *m*_*j*_ only using the *j*-th seismic trace record *d*_*j*_ independently which totally ignores the correlation with other sampled data ${d}_{\hat{j}}$ ($\hat{j}\ne j$). Generally, multiple seismic wavefield traces are related to each other. The similarity and difference hidden in the given group of seismic traces are expected to improve the recovery performance. To detailedly demonstrate the correlationship among multiple seismic traces, we randomly choose three trace observations from a seismogram generated from a seven layers geologic velocity model (see Experimental Section for details). Two neighboring traces are denoted by *d*_1_ and *d*_2_ while the third one *d*_3_ is relatively far from them. We separately recover the representation coefficients ${m}_{j}^{\ast },j=1,2,3$ by solving

5 $${m}_{j}^{\ast }=\text{arg}\mathop{\min }\limits_{{m}_{j}\in {R}^{K}}J({m}_{j})=\parallel L{m}_{j}-{d}_{j}{\parallel }_{2}^{2}+\alpha {\sum _{k=1}^{K}(|{m}_{j}^{k}|)}^{p},\quad j=1,2,3,p=0.5,$$

where ${m}_{j}^{k}$ is *k*-th entry of *m*_*j*_. The weight values of recovered coefficients are plotted in Fig. 1(a–c). The horizontal axis denotes the coordinates of the representation vector while the vertical axis shows the weight quantities of representation coefficients, namely $|{({m}_{j}^{\ast })}^{k}|,k=1,2,\cdots ,256;j=1,2,3$. The curves clearly display the similar clustering and sparse pattern of three recovered coefficients. The correlations inspire us to assume that the multiple traces coefficients share the same distribution. For comparison, we jointly recover three coefficients simultaneously from *D*_1, 2, 3_ = [*d*_1_, *d*_2_, *d*_3_] $\in$ *R*^*m* × 3^ by a matrix minimization problem

6 $${M}_{1,2,3}^{\ast }=\text{arg}\mathop{\min }\limits_{{M}_{1,2,3}\in {R}^{K\times 3}}J({M}_{1,2,3})=\parallel L{M}_{1,2,3}-{D}_{1,2,3}{\parallel }_{F}^{2}+\alpha {\sum _{k=1}^{K}(\parallel {m}_{1,2,3}^{k}{\parallel }_{2})}^{p},\,\,p=0.5,$$

where ${M}_{1,2,3}^{\ast }\in {R}^{K\times 3}$ and ${m}_{1,2,3}^{k}\in {R}^{3}$ is the *k*-th row of *M*_1, 2, 3_. Since three vector minimizations as (5) are integrated to a matrix one (6), each entry ${m}_{j}^{k}$ of representative vector is spanned to a row vector ${m}_{1,2,3}^{k}\in {R}^{3}$. Hence the absolute values of weight entries in (5) are naturally generalized to *l*_2_ norm of row vector for its smoothness, that is $|{m}_{j}^{k}|\to \parallel {m}_{1,2,3}^{k}{\parallel }_{2}$. To illustrate the jointly recovered coefficient matrix ${M}_{1,2,3}^{\ast }$ of (6) also follows the similar variation as in Fig. 1(a–c), we measure the *l*_2_ norm of each row vector in the joint sense corresponding to $|{({m}_{j}^{\ast })}^{k}|$,

7 $$|{({m}_{j}^{\ast })}^{k}|\to {\parallel {({M}_{1,2,3}^{\ast })}^{k}\parallel }_{2}={({|{({m}_{1,2,3}^{\ast })}_{1}^{k}|}^{2}+{|{({m}_{1,2,3}^{\ast })}_{2}^{k}|}^{2}+{|{({m}_{1,2,3}^{\ast })}_{3}^{k}|}^{2})}^{\frac{1}{2}},k=1,2,\cdots ,256.$$

Clearly, the joint representation coefficients also exhibit similar sparse pattern and weight concentration to the individual models (see Fig. 1(d)).

Under the assumption that multiple seismic wavefield traces jointly share the similar weight parameter pattern, we propose to express all the sub-sampled observations over the same bases simultaneously as

8 $$LM=D,$$

where *D* = [*d*_1_, *d*_2_, …, *d*_*l*_] is composed of *l* seismic observations and *M* = [*m*_1_, *m*_2_, …, *m*_*l*_] denotes the corresponding coefficient matrix. As far as the columns are concerned, the equation (8) is an easy consequence of the equation (3). Figure 1 has demonstrated that the multiple seismic traces are related to each other, especially when the samples are obtained in the similar fields. We reasonably measure the joint compactness and correlation of the multiple observations in row sense. By reviewing *l*_*q*_−*l*_*p*_(*q* > 0, *p* > 0) model (4), we notice that the expression errors *e*_*j*_ = *Lm*_*j*_−*d*_*j*_, *j* = 1, 2, …, *l* and the priori of representation coefficients are assumed to submit to the independent identically distribution,

9 $$P({e}_{j}|{m}_{j})\propto \underset{k=1}{\overset{K}{{\rm{\Pi }}}}\exp (-{|{e}_{j}^{k}|}^{q}),P({m}_{j})\propto \underset{k=1}{\overset{K}{{\rm{\Pi }}}}\exp (-{\alpha }_{k}{|{m}_{j}^{k}|}^{p}),{\alpha }_{k} > 0$$

where ${m}_{j}^{k}$ is the *k*-th entry of representation vector *m*_*j*_ $\in$ *R*^*K*^. The solution ${m}_{j}^{\ast }$ to (4) can be rewritten as the maximum likelihood estimation

10 $${m}_{j}^{\ast }=\text{arg}\mathop{\max }\limits_{{m}_{j}\in {R}^{K}}\,\mathrm{ln}\,P({m}_{j}|{e}_{j})=\text{arg}\mathop{\max }\limits_{{m}_{j}\in {R}^{K}}(\mathrm{ln}\,P({e}_{j}|{m}_{j})+\,\mathrm{ln}\,P({m}_{j}))$$

Because each coefficient component ${m}_{j}^{k}$ in (3) is spanned to a row vector in the joint expression system (8), the absolute value of the scalar component is naturally replaced by a vector norm. Euclidean norm is preferred for its smoothness and easiness. Based on the analysis (9) and (10), the joint sparse priori of coefficient matrix *M* and fidelity error matrix *E* = *LM*−*D* can be considered

$$P(E|M)\propto \underset{k=1}{\overset{K}{{\rm{\Pi }}}}\exp (-{\Vert {e}^{k}\Vert }_{2}^{q}),P(M)\propto \underset{k=1}{\overset{K}{{\rm{\Pi }}}}\exp (-{\alpha }_{k}{\Vert {m}^{k}\Vert }_{2}^{p}),{\alpha }_{k} > 0$$

where *m*^*k*^, *e*^*k*^ are the *k*-th row vectors of *M* $\in$ *R*^*k* × *l*^ and *E* $\in$ *R*^*m* × *l*^ respectively.*α*_*k*_ > 0 is a constant and ${\Vert .\Vert }_{2}$ stands for the Euclidean norm. In the similar relationship between (4) and (9), the joint matrix minimization approach for the ill-posed linear system (8) can be generally formulated as

11 $$\mathop{\min }\limits_{M}\,J(M)=\parallel LM-D{\parallel }_{2,q}^{q}+\parallel {\rm{\Lambda }}M{\parallel }_{2,p}^{p},\,\,\,\,\,\,\,\,q > 0,p > 0,$$

where the *l*_2, *p*_ norm of the priori matrix *M* is defined as

12 $$\parallel M{\parallel }_{2,p}^{p}=\sum _{k=1}^{K}\parallel {m}^{k}{\parallel }_{2}^{p},\,\,\,\,p\in (0,1].$$

Here $\parallel LM-D{\parallel }_{2,q}(q > 0)$ denotes the *l*_2, *q*_ matrix norm of *LM*−*D*, ${\rm{\Lambda }}=diag{\{{\alpha }_{k}\}}_{k=1}^{K}$ is a regularization matrix and its diagonal entry *α*_*k*_ > 0 is the regularization parameter for the *k*-th row of *M*. Especially, if *M* contains only one column *m*_*j*_, each $\parallel {m}^{k}{\parallel }_{2}$ is reduced to $|{m}_{j}^{k}|$ while $\parallel M{\parallel }_{2,p}$ is equivalent to $\parallel {m}_{j}{\parallel }_{p}$. When Λ takes scalar identity, the joint system (11) is exactly reduced to (4).

There are different choices of the parameter pair *q* > 0 and *p* > 0. Here we are interested in *q* = 2 and *p* $\in$ (0, 1] for the practical purpose. Extensive studies have illustrated that the fractional norm *l*_*p*_ (*p* $\in$ (0, 1)) has better sparsity than *l*_1_ norm^35–39^. But the *l*_*p*_ norm is neither Lipschitz nor convex which brings computational challenge. This paper presents a unified algorithm to solve the mixed *l*_2, *p*_ regularized matrix minimization problem (11) for any *p* $\in$ (0, 1]. The computational results in seismic wavefield recovery validate the efficient performance of the joint matrix minimization approach. The convergence properties of our new algorithm are also analyzed.

## Algorithms

In this section, a unified method will be developed to solve the *l*_2, *q*_−*l*_2, *p*_ matrix minimization problem for any *q* = 2 and 0 < *p* ≤ 1. Especially when *p* is fractional, (11) is neither convex nor Lipschitz continuous which brings many computational difficulties. Actually the unconstrained *l*_*q*_-*l*_*p*_ minimization is strongly NP-hard for any 0 < *q* or *p* < 1^40^. Reweighed minimization algorithm^35,41,42^ is an efficient algorithm for solving the *l*_2_-*l*_*p*_ (0 < *p* < 1) vector minimization problem which has been extended by Wang *et al*.^43^ to solve matrix minimization problem. Even the problem considered in^43^ is the special case of (11) with *q* = *p* $\in$ (0, 1], the idea motivates us to develop an iteratively quadratic algorithm for the generalized *l*_2, *p*_ matrix minimization for *p* $\in$ (0, 1]. Moreover, the convergence analysis will be uniformly demonstrated.

After simple transformation, $\parallel {\rm{\Lambda }}M{\parallel }_{2,p}^{p}$ can be rewritten as

13 $$\parallel {\rm{\Lambda }}M{\parallel }_{2,p}^{p}=Tr({M}^{T}HM),$$

where $Tr(\cdot )$ stands for the trace operation and

14 $$H={\rm{diag}}\{\frac{{\alpha }_{1}}{\parallel {m}^{1}{\parallel }_{2}^{2-p}},\frac{{\alpha }_{2}}{\parallel {m}^{2}{\parallel }_{2}^{2-p}},\cdots ,\frac{{\alpha }_{K}}{\parallel {m}^{d}{\parallel }_{2}^{2-p}}\},$$

where *m*^*k*^ (*k* = 1, 2, …, *K*) is the *k*-th row vector of *M*.

Hence the objective function of (11) for *q* = 2, *p* $\in$ (0, 1] can be reformulated as

15 $$\begin{array}{c}J(M):={\Vert LM-D\Vert }_{2,2}^{2}+{\Vert {\rm{\Lambda }}M\Vert }_{2,p}^{p}\\ \,\,\,\,\,\,=Tr({(LM-D)}^{T}(LM-D))+Tr({M}^{T}HM)\end{array}$$

It is well known that the *KKT* point of the unconstrained optimization problem (11) is also the stationary point of *J*(*M*)^44^. Compute the derivative of *J*(*M*) with respect to matrix *M* and set it to zero, we get the *KKT* equation of the problem (11) as follows

16 $$\frac{\partial J(M)}{\partial M}=2{L}^{T}(LM-D)+pHM=0.$$

Thus solving (11) is reduced to finding the solution of the nonlinear equation (16). If *H* is fixed and the matrix $N={L}^{T}L+\frac{p}{2}H$ is invertible, equation (16) can be solved by

17 $$M={({L}^{T}L+\frac{p}{2}H)}^{-1}{L}^{T}D.$$

We notice that if some row of *M* is zero, the diagonal entries of *H* cannot be generated, nor can *N*. Then the iteration breaks down. In view of the seismic wavefield recovery, the zero row means the corresponding basis function has no contribution to reconstruct all the observed seismic traces. For example, if *m*^*k*^ = 0, then *L*_*k*_ (the *k*-th column of transformation matrix *L*) is nothing with the observations *D* in the representation system (8). To avoid the possible breakdown of the matrix *N* in (17) and reasonably explain this numerical behavior, we apply the Sherman-Morrison-Woodbury formula^45^ to *N*^−1^. Denote

18 $$G={(\frac{p}{2}H)}^{-1}=\frac{2}{p}{\rm{diag}}\{\frac{\parallel {m}^{1}{\parallel }_{2}^{2-p}}{{\alpha }_{1}},\frac{\parallel {m}^{2}{\parallel }_{2}^{2-p}}{{\alpha }_{2}},\cdots ,\frac{\parallel {m}^{K}{\parallel }_{2}^{2-p}}{{\alpha }_{K}}\},$$

then the formula (17) can be rewritten as

19 $$M={N}^{-1}{L}^{T}D=[G-G{L}^{T}{({I}_{m}+LG{L}^{T})}^{-1}LG]{L}^{T}D,$$

where *I*_*m*_ is *m*-dimensional identity operator. If matrices *G* and *M* are computed alternatively corresponding to equations (18) and (19) respectively, then an iterative procedure can be naturally developed

20 $$\begin{array}{l}{G}_{t}=\frac{2}{p}diag\{\frac{\parallel {m}_{t}^{1}{\parallel }_{2}^{2-p}}{{\alpha }_{1}},\frac{\parallel {m}_{t}^{2}{\parallel }_{2}^{2-p}}{{\alpha }_{2}},\cdots ,\frac{\parallel {m}_{t}^{K}{\parallel }_{2}^{2-p}}{{\alpha }_{K}}\},\\ {M}_{t+1}=[{G}_{t}-{G}_{t}{L}^{T}{({I}_{m}+L{G}_{t}{L}^{T})}^{-1}L{G}_{t}]{L}^{T}D.\end{array}$$

The iterative algorithm is outlined in Algorithm 1.

**Algorithm 1**. An iterative procedure for solving problem (16)

Step 1. Input *L* $\in$ *R*^*m* × *K*^, *D* $\in$ *R*^*m* × *l*^. Set the sparse parameter *p* $\in$ (0, 1] and diagonal matrix ${\rm{\Lambda }}=diag\{{\alpha }_{1},{\alpha }_{2},\cdots ,{\alpha }_{K}\}\,\succ \,0$ (here $\succ$ refers to the positive definite). Given the stopping criterion $\epsilon  > 0$.

Step 2. Set *t* = 1 and initialize *M*_1_ $\in$ *R*^*K* × *l*^.

Step 3. For *t* = 1, 2, … until ${\rho }_{t}\le \epsilon$ do:

$${G}_{t}=\frac{2}{p}diag\{\frac{\parallel {m}_{t}^{1}{\parallel }_{2}^{2-p}}{{\alpha }_{1}},\frac{\parallel {m}_{t}^{2}{\parallel }_{2}^{2-p}}{{\alpha }_{2}},\cdots ,\frac{\parallel {m}_{t}^{K}{\parallel }_{2}^{2-p}}{{\alpha }_{K}}\};$$

$${M}_{t+1}=[{G}_{t}-{G}_{t}{L}^{T}{({I}_{m}+L{G}_{t}{L}^{T})}^{-1}L{G}_{t}]{L}^{T}D;$$

$${\rho }_{t}=\frac{\parallel {M}_{t+1}-{M}_{t}{\parallel }_{F}}{\parallel {M}_{t}{\parallel }_{F}}.$$

The ${m}_{t}^{k}$ (*k* = 1, 2, …, *K*) means the *k*-th row vector of *M*_*t*_. Algorithm 1 aims to solve the fixed-point system (16) which is the stationary equation of the matrix function (15). Based on the iterative procedure of Algorithm 1, the iterative point *M*_*k*_ is the solution of the nonlinear equation (16) if and only if *M*_*t*_ = [*G*_*t*_−*G*_*t*_*L*^*T*^(*I*_*m*_ + *LG*_*t*_*L*^*T*^)^−1^*LG*_*t*_]*L*^*T*^*D* which is equivalent to *M*_*k*_ = *M*_*k* + 1_. From this iteration on, the iteration point will not update which indicates that a stationary point has been found. Hence the stopping criterion of Algorithm 1 can be chosen as ${\rho }_{t}:=\frac{\parallel {M}_{t+1}-{M}_{t}{\parallel }_{F}}{\parallel {M}_{t}{\parallel }_{F}}\le \epsilon$, where $\parallel \cdot {\parallel }_{F}$ stands for the Frobenius norm^46^.

Based on the definition (12) of $\parallel M{\parallel }_{2,p}$, the sparse parameter *p* $\in$ (0, 1] aims to find a solution with many zero row vectors of the *l*_2, *p*_-regularized matrix minimization problem (11). This means that many basis functions have no contribution to reconstruct the seismic wavefields which accords with the prior knowledge. Therefore (*m*_*t*_)^*k*^ = 0 might frequently occur during the iterations of Algorithm 1. We may formulate the following statement.

**Remark**. In Algorithm 1, if ${m}_{{t}_{0}}^{k}=0$ happens for some iteration ${M}_{{t}_{0}}$, then ${m}_{t}^{k}=0$ for *t* ≥ *t*_0_.

We give explanations of the above remark as follow. If ${m}_{{t}_{0}}^{k}=0$ in the *t*_0_-th iteration, then the diagonal entry of ${G}_{{t}_{0}}$ is zero, namely ${({G}_{{t}_{0}})}_{kk}=0$. From the update formula ${M}_{{t}_{0}+1}={G}_{{t}_{0}}[{I}_{K}-{L}^{T}{({I}_{m}+L{G}_{{t}_{0}}{L}^{T})}^{-1}L{G}_{{t}_{0}}]{L}^{T}D$, we know that ${m}_{{t}_{0}+1}^{k}=0$ holds, so does ${m}_{t}^{k}=0$ for *t* ≥ *t*_0_. After *t*_0_ iterations with ${m}_{{t}_{0}}^{k}=0$, the *k*-th column of the matrix *L* is unnecessary in the linear system (8) and the variational function *J*(*M*) in (15). So we can discard the *k*-th column of the matrix *L* to reduce the system without any loss. The improvement of Algorithm 1 can be concluded as Algorithm 2.

**Algorithm 2**. Solving problem (16) for any *p* $\in$ (0, 1]

Step 1. Input *L* $\in$ *R*^*m* × *K*^, *D* $\in$ *R*^*m* × *l*^. Set the sparse parameter *p* $\in$ (0, 1] and the diagonal matrix ${\rm{\Lambda }}=diag\{{\alpha }_{1},{\alpha }_{2},\cdots ,{\alpha }_{K}\}\,\succ \,0$. Given stopping criterion $\epsilon  > 0$.

Step 2. Set *t* = 1 and initialize ${\hat{M}}_{1}\in {R}^{K\times l}$. Let Ω_0_ = {1, 2, …, *K*}.

Step 3. For *t* = 1, 2, … until ${\rho }_{t}\le \epsilon$ do:

$${{\rm{\Omega }}}_{t}={{\rm{\Omega }}}_{t-1}\setminus \{k:\,\parallel {\hat{m}}_{t}^{k}{\parallel }_{2}=0\};$$

$${M}_{t}={\hat{M}}_{t}({{\rm{\Omega }}}_{t};:),\,{L}_{t}=L(:;{{\rm{\Omega }}}_{t});$$

$${G}_{t}=\frac{2}{p}diag{\{\frac{\parallel {m}_{t}^{k}{\parallel }_{2}^{2-p}}{{\alpha }_{k}}\}}_{k\in {{\rm{\Omega }}}_{t}};$$

$${\hat{M}}_{t+1}=[{G}_{t}-{G}_{t}{L}_{t}^{T}{({I}_{m}+{L}_{t}{G}_{t}{L}_{t}^{T})}^{-1}{L}_{t}{G}_{t}]{L}_{t}^{T}D;$$

$${\rho }_{t}=\frac{\parallel {\hat{M}}_{t+1}-{M}_{t}{\parallel }_{F}}{\parallel {M}_{t}{\parallel }_{F}}.$$

In Algorithm 2, ${M}_{t}={\hat{M}}_{t}({{\rm{\Omega }}}_{t};:)$ means to keep the rows of ${\hat{M}}_{t}$ corresponding to the index set Ω_*t*_ while *L*_*t*_ = *L*(:;Ω_*t*_) keeps the column of *L* corresponding to Ω_*t*_. Compared with Algorithm 1, Algorithm 2 removes the zero rows of the approximation solution in each iteration and the corresponding columns of the bases matrix *L*. This technique iteratively reduces the inactive set of data.

Based on the procedure of Algorithm 2, ${N}_{t}={L}_{t}^{T}{L}_{t}+\frac{p}{2}{H}_{t}$ is well defined and ${\hat{M}}_{t+1}$ is the solution of the linear system ${N}_{t}M={L}_{t}^{T}D$. Since *N*_*t*_ is symmetric and positive definite, ${\hat{M}}_{t+1}$ is also the optimal matrix solution of the following quadratic subproblem

21 $$\mathop{\min }\limits_{M}\,{Q}_{t}(M):=Tr({({L}_{t}M-D)}^{T}({L}_{t}M-D))+Tr({M}^{T}HM).$$

We would have ${Q}_{t}({\hat{M}}_{t+1})\le {Q}_{t}({M}_{t})$, which is equivalent to

22 $$\parallel {L}_{t}{\hat{M}}_{t+1}-D{\parallel }_{F}^{2}+\frac{p}{2}\sum _{k\in {{\rm{\Omega }}}_{t}}\frac{{\alpha }_{k}\parallel {\hat{m}}_{t+1}^{k}{\parallel }_{2}^{2}}{\parallel {m}_{t}^{k}{\parallel }_{2}^{2-p}}\le \parallel {L}_{t}{M}_{t}-D{\parallel }_{F}^{2}+\frac{p}{2}\parallel {{\rm{\Lambda }}}_{t}{M}_{t}{\parallel }_{2,p}^{p}.$$

It is noticed that $J({M}_{t})=\parallel {L}_{t}{M}_{t}-D{\parallel }_{F}^{2}+\parallel {{\rm{\Lambda }}}_{t}{M}_{t}{\parallel }_{2,p}^{p}$ and $J({M}_{t+1})=J({\hat{M}}_{t+1})$. Using inequalities (A-2) (see the Appendix A) and (22), we can derive that

23 $$\begin{array}{l}J({M}_{t+1})=J({\hat{M}}_{t+1})\le J({M}_{t}),\,\,\,\,p\in (0,1],\end{array}$$

which means {*J*(*M*_*t*_)} will decrease with respect to iterations for any *p* $\in$ (0, 1].

Once *J*(*M*_*t* + 1_) = *J*(*M*_*t*_) happens for some *t*, the equalities in (A-2) (see the Appendix A) and (22) hold simultaneously. From Proposition 2 of the Appendix A, we obtain $\parallel {\hat{m}}_{t+1}^{k}{\parallel }_{2}=\parallel {m}_{t}^{k}{\parallel }_{2}$ for all *k* $\in$ Ω_*t*_. Thus *G*_*t* + 1_ = *G*_*t*_ and *H*_*t* + 1_ = *H*_*t*_, which implies that ${\hat{M}}_{t+1}$ is a solution of the equation (17). Since the objective function sequence {*J*(*M*_*t*_)} for all *t* is strictly decreasing and lower bounded, any accumulation of the set {*M*_*t*_} is a stationary point of the equation (11). At the same time, the descending quantity of {*J*(*M*_*t*_)} measures the convergence precision of the matrix sequence {*M*_*t*_}.

Once the nonzero set of the *t*-th iteration has been fixed, the subproblem (21) can be solved in a variety of ways such as preconditioned conjugate gradient methods^46^, nonmonotone gradient descent methods^47,48^, and so on. The framework can be concluded as Algorithm 3.

**Algorithm 3**. A unified algorithm for solving problem (16) for any *p* $\in$ (0, 1]

Step 1. Input *L* $\in$ *R*^*m* × *K*^, *D*${\rm{\Lambda }}=diag\{{\alpha }_{1},{\alpha }_{2},\cdots ,{\alpha }_{K}\}\,\succ \,0$ $\in$ *R*^*m* × *l*^. Set the sparse parameter *p* $\in$ (0, 1] and the diagonal matrix . Given stopping criterion $\epsilon  > 0$.

Step 2. Set *t* = 1 and initialize ${\hat{M}}_{1}\in {R}^{K\times l}$. Let Ω_0_ = {1, 2, …, *K*}.

Step 3. For *t* = 1, 2, … until ${\rho }_{t}\le \epsilon$ do:

$${{\rm{\Omega }}}_{t}={{\rm{\Omega }}}_{t-1}\setminus \{k|\parallel {\hat{m}}_{t}^{k}{\parallel }_{2}=0\};$$

$${M}_{t}={\hat{M}}_{t}({{\rm{\Omega }}}_{t};:);\,{L}_{t}=L(:;{{\rm{\Omega }}}_{t});$$

$${H}_{t}={\rm{diag}}{\{\frac{{\alpha }_{k}}{\parallel {m}_{t}^{k}{\parallel }_{2}^{2-p}}\}}_{k\in {{\rm{\Omega }}}_{t}};$$

$${N}_{t}={L}_{t}^{T}{L}_{t}+\frac{p}{2}{H}_{t};$$

Solve ${N}_{t}M={L}_{t}^{T}D$ for the solution ${\hat{M}}_{t+1}$;

$${\rho }_{t}=\frac{\parallel {\hat{M}}_{t+1}-{M}_{t}{\parallel }_{F}}{\parallel {M}_{t}{\parallel }_{F}}.$$

## Experimental results

To validate the efficiency of the joint matrix minimization approach and the unified algorithm for the problem (11), we perform three tests: (1) restoration of the input one-dimensional random signal with the randomly generated matrix *L*; (2) restoration of the synthetic seismic data with random loss of traces; (3) restoration of the field data.

### One-dimensional signal reconstruction

We randomly take samples to generate the matrix *L*. For implementation, we try to restore the signal by the model (11) with *q* = 2 and *p* $\in$ (0, 1].

The stopping precision in Algorithm 3 is set to $\epsilon ={10}^{-3}$. The sparse parameter *p* and regularization parameter *α*_*k*_ are typically chosen in (0, 1]. Results for other values of *p* are similar. The relative error of the recovered signal *M*_rec_ to the true (given) signal *M*_true_ is defined by

$${{\rm{err}}}_{{\rm{rel}}}=\frac{\parallel {M}_{{\rm{rec}}}-{M}_{{\rm{true}}}{\parallel }_{2}}{\parallel {M}_{{\rm{true}}}{\parallel }_{2}}.$$

To quantify the results, we define the signal-to-noise ratio (SNR) as ${\rm{SNR}}=10{\mathrm{log}}_{10}\frac{\parallel {d}_{{\rm{org}}}{\parallel }_{2}^{2}}{\parallel {d}_{{\rm{org}}}-{d}_{{\rm{rec}}}{\parallel }_{2}^{2}}$, where *d*_org_ refers to the original data and *d*_rec_ is the restored data.

For the one-dimensional case, the matrix *M* is reduced to a vector, hence the unified Algorithm 3 can be used for solving (11). For comparison, we also apply spectral projected gradient (SPG) method^49^ to solve the *l*_1_-regularization problem. The code of SPG is downloaded from http://www.cs.ubc.ca/~mpf/spgl1/index.html. Two algorithms are carried out in the same environment and choose their best regularization parameters. The comparison items include err_rel_ value, SNR and CPU running time (second). Each experiment is repeated five times and the average values are reported in Table 1. It indicates that both methods perform well for one-dimensional signal reconstruction problem.

**Table 1:** The experimental results of one-dimensional seismic wavefield reconstruction.

Methods	No noise			Noise level 0.001			Noise level 0.01		
	err_rel_	SNR	CPU(s)	err_rel_	SNR	CPU(s)	err_rel_	SNR	CPU(s)
Alg.3 (p = 0.5)	2.56e-4	71.83	4.56e-2	3.54e-4	69.02	1.7e-2	2.09e-2	33.62	3.25e-2
Alg.3 (p = 1)	5.08e-4	65.89	3.18e-2	6.67e-4	63.51	1.81e-2	2.48e-2	32.12	3.09e-2
SPG (p = 1)	2.62e-4	71.07	5.56e-2	3.75e-4	68.96	4.22e-2	2.54e-2	31.89	6.54e-2

**Figure 2:**
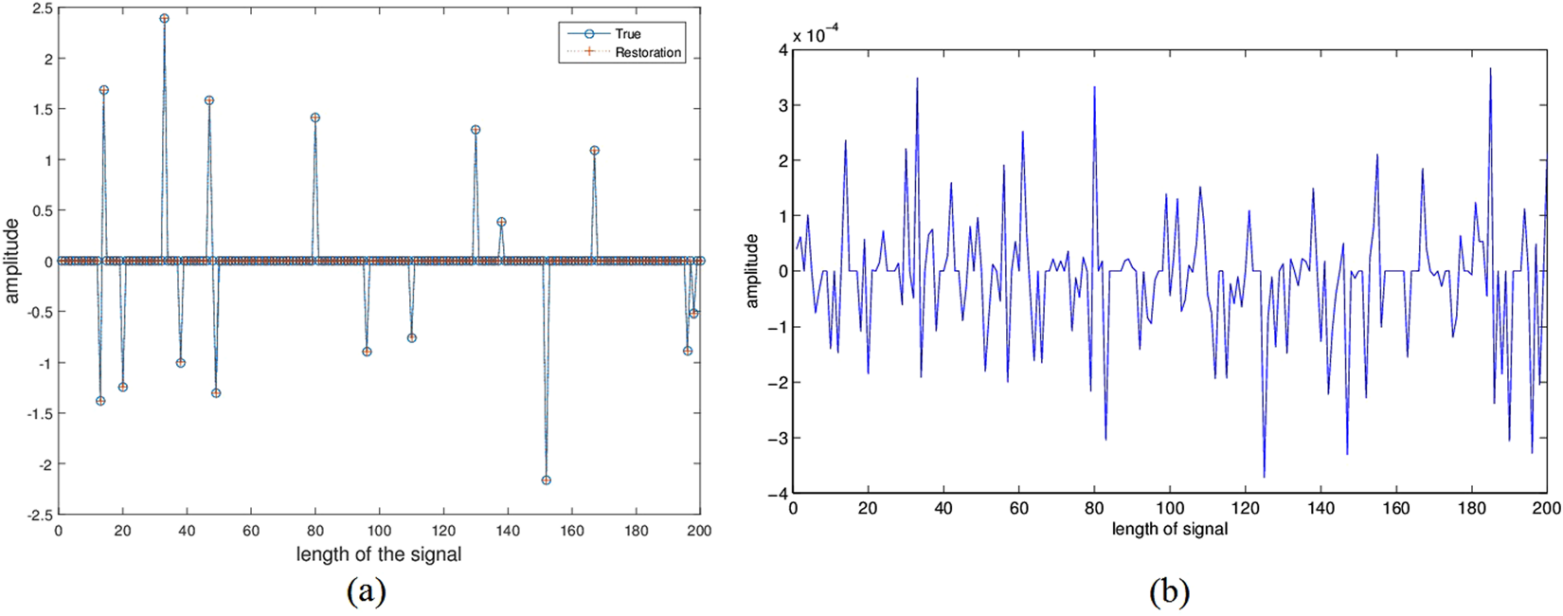
One-dimensional experimental results via model (11) with *q* = 2 and $p=1/2$ for noisy data: (**a**) comparison of the original and the recovered signal; (**b**) difference between the recovered signal and the original signal.

Apart from the regular data, we also consider the noisy cases to show the robustness of two methods. Different noise levels are added to the simulated data. Noise level 0.001 means the noise is randomly generated with zero mean and 0.001 variance. The results of Algorithm 3 with sparse parameters *p* = 1 and *p* = 0.5 are displayed in Table 1. Compared with the *l*_1_-regularized minimization model, the half-norm regularized minimization behaves better in reconstruction. Figure 2 plots the recovery performance of the Algorithm 3 with *p* = 0.5 on noisy data. Figure 2(a) is the comparison of the real signal and the recovered signal, Fig. 2(b) illustrates the difference between the recovered signal and the input (true) signal. The recovery images of other cases are similar. The figures reveal that our model and algorithm perform well for one-dimensional seismic wavefield reconstruction problem even in noisy cases.

### Reconstruction of seismograms from a layered model

Now we consider a seismogram generated from a seven layers geologic velocity model where the spatial sampling interval is 15 meters and the time sampling interval is 0.002 second. The velocity varies from 2500 m/s to 5500 m/s. The seismogram is generated using a source function given by a Ricker wavelet with central-frequency of 25 Hz. The dataset contains 256 traces with 256 time samples in each trace. Different percentages of missing traces in original data, 10%, 25% and 50%, are used to test the limitation of recovery methods. The joint matrix model (11) with Algorithm 3 is applied to reconstruct the seismic wavefield. Since the spectral projected gradient method only solves an *l*_1_-regularized vector minimization problem, we decompose the matrix representation system (11) into the *l*_1_-regularized vector minimization problem. Each column is considered as a subproblem to reconstruct its weight vector separately. Then all the solutions of the subproblems are sequentially aligned into a weighted matrix to evaluate the reconstruction performance. The experimental results on missing percentages 10% and 25% can be seen in Tables 2 and 3.

**Table 2:** Two-dimensional seismic wavefield reconstruction on 10% missing data.

Methods	No noise			Noise level 0.001		
	err_rel_	SNR	CPU(s)	err_rel_	SNR	CPU(s)
Alg.3 (p = 0.1)	0.0063	44.0585	0.326	0.0098	40.2009	0.5056
Alg.3 (p = 0.2)	0.0075	42.5209	0.3277	0.0092	40.7235	0.36
Alg.3 (p = 0.5)	0.0112	39.049	0.4796	0.0122	38.308	0.5044
Alg.3 (p = 1)	0.0115	38.7591	0.135	0.0121	38.3405	0.0973
SPG (p = 1)	0.0711	22.9599	13.121	0.0726	22.7818	12.9125

**Table 3:** Two-dimensional seismic wavefield reconstruction on 25% missing data.

Methods	No noise			Noise level 0.001		
	err_rel_	SNR	CPU(s)	err_rel_	SNR	CPU(s)
Alg.3 (p = 0.1)	0.0204	33.8269	0.2916	0.0209	33.5817	0.2451
Alg.3 (p = 0.2)	0.0179	34.9441	0.4101	0.0252	31.9641	0.4695
Alg.3 (p = 0.5)	0.0219	33.2043	0.3775	0.0283	30.9583	0.4783
Alg.3 (p = 1)	0.0314	30.075	0.0962	0.0346	29.228	0.0894
SPG (p = 1)	0.0953	20.4223	25.7615	0.0953	20.4138	21.894

As for the data without noise but missing 50% traces, the reconstruction performance of joint matrix model with Algorithm 3 is much worse than missing percentages of 10% and 25%. The err_rel_ value is 0.5414 and SNR is around 5.1904dB, almost the same for any *p* $\in$ (0, 1]. These results mean that our method may not completely recover the seismic wavefield well if the missing trace signals are more than 50%. Actually, the sub-sampled data missing 50% itself is a failed collection of seismic recodes.

**Figure 3:**
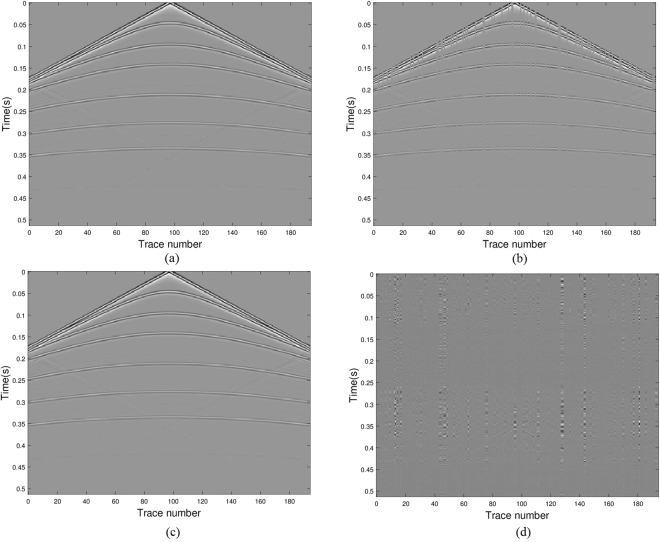
Seismic data results via model (11) with *q* = 2 and *p* = 0.5: (**a**) the real data; (**b**) the data with missing traces; (**c**) the recovered data; (**d**) error between the original and the recovered signals.

The original shot gathers are shown in Fig. 3(a). The data with 25% traces missing are shown in Fig. 3(b). In forming the under-determined matrix *L*, a Haar wavelet orthogonal base is used to form the transform matrix Ψ. The unified Algorithm 3 is applied to solve the joint matrix minimization problems (11) with *q* = 2 and typical parameters *p* $\in$ (0, 1]. Good recovery performance is observed and the result is demonstrated in Fig. 3(c). The error of the original and the recovered data shown in Fig. 3(d) illustrates the efficient recovery performance of joint matrix minimization approach. In displaying the results, the amplitude scale of the error map is the same as the amplitude scale of the data. Of course, other values of the sparse parameter *p* can be chosen, the results in visualization are similar. So, we only list the quantitative results in Tables 2 and 3.

**Figure 4:**
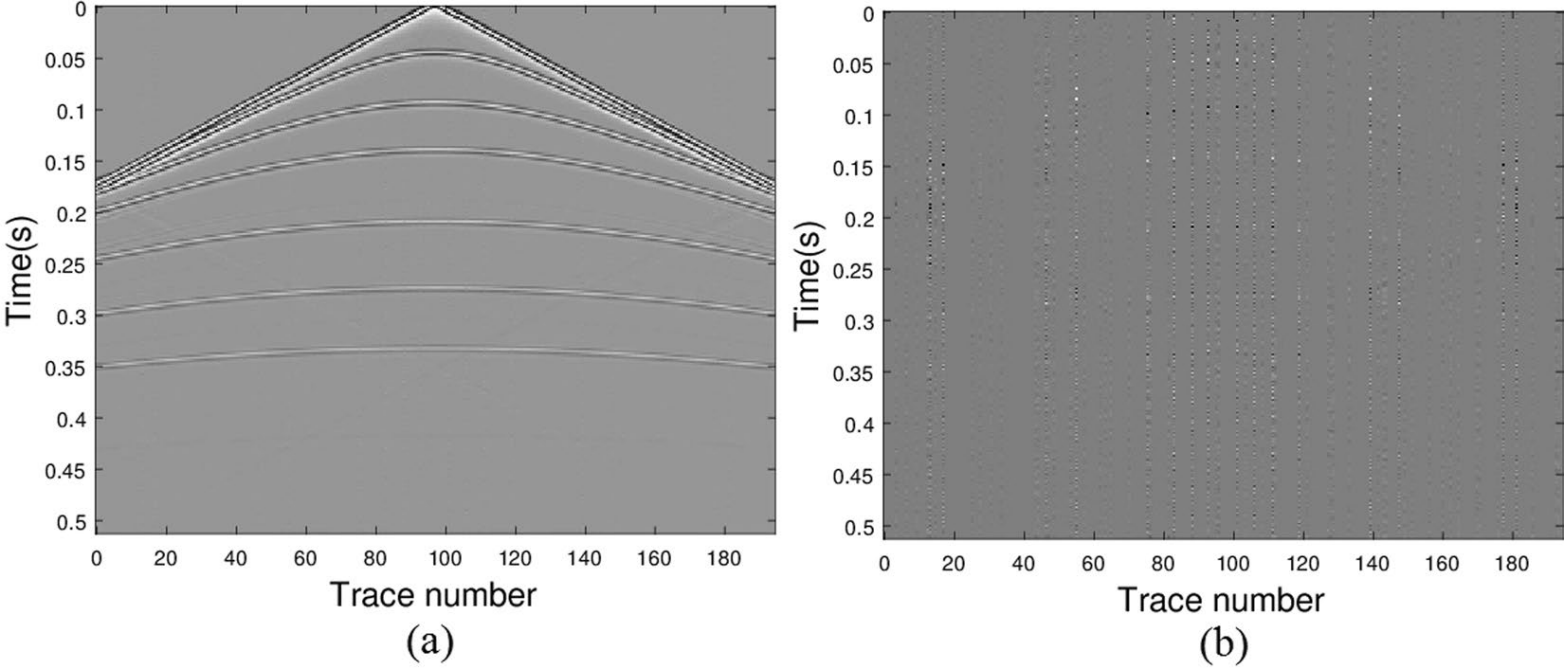
Seismic data results via SPG for *l*_1_-regularized least square minimization: (**a**) the recovered data; (**b**) error between the original and the recovered signals.

Comparatively, the recovery image of the SPG algorithm for the case of 25% traces missing is presented in Fig. 4. Figure 4(a) is the reconstruction and Fig. 4(b) displays the difference between the original and reconstructed seismic signals. It is noticed that SPG algorithm for the *l*_1_-regularization vector minimization restores the seismic wavefield as accurate as the joint matrix approach with Algorithm 3. These results are obtained using the same code from http://www.cs.ubc.ca/~mpf/spgl1/index.html.

**Figure 5:**
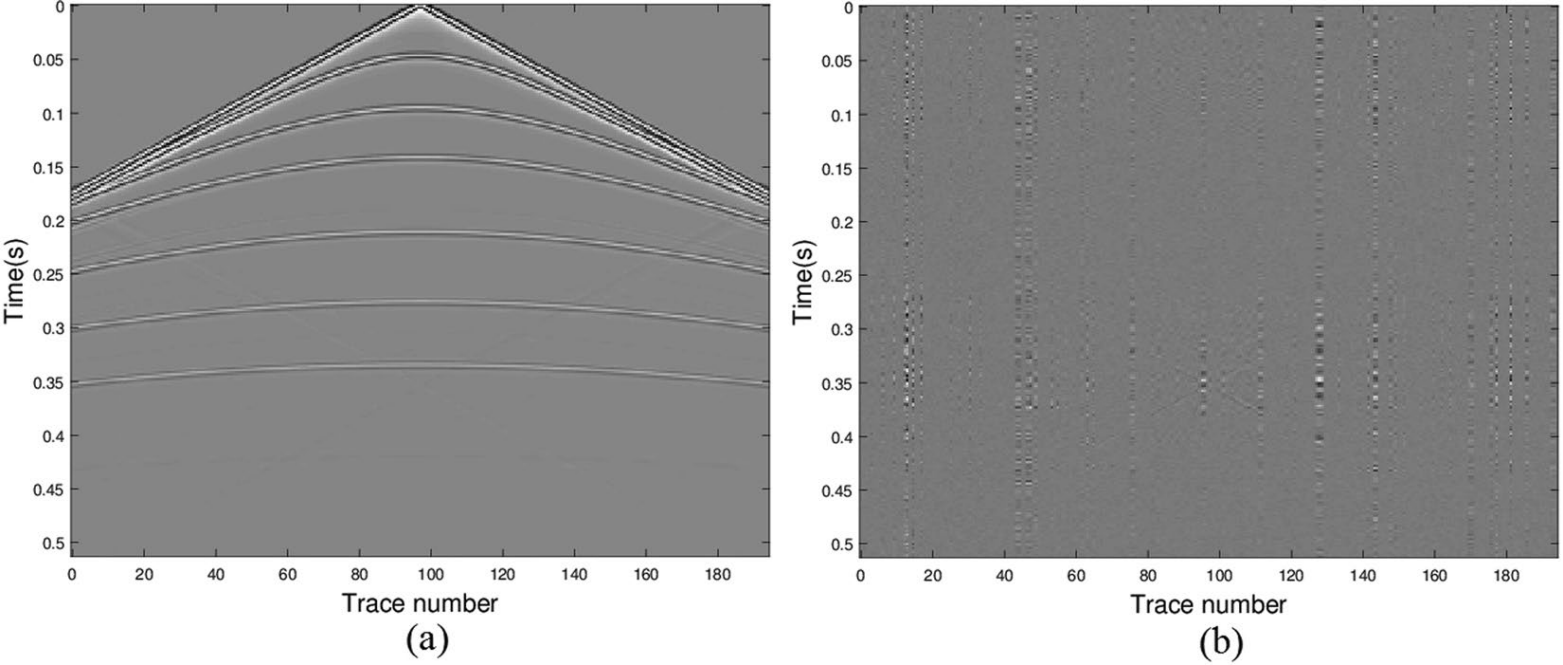
Seismic data results in noisy case via model (11) with *q* = 2 and *p* = 0.5: (**a**) the recovered data; (**b**) error between the original and the recovered signals.

To show the anti-noise property of our algorithm, we add random noise with noise level 0.001 to the simulated data. The unified Algorithm 3 is applied to solve the joint matrix minimization problems. The err_rel_ value, SNR and CPU running time (second) are listed in Table 2 for 3 sparse parameters. The recovery image and the error of the original and the recovered data are shown in Fig. 5(a and b) respectively. The low relative error and high SNR indicate that our algorithm is stable for seismic data restoration.

To save memory requirement of large-scale data, we have observed the restoration behavior of our method on patch of the input synthetic data. We evenly partition the collection of trace signals *D* into several blocks, such as *D* = [*D*_1_, *D*_2_, …, *D*_*f*_], where ${D}_{g}\in {R}^{m\times {l}_{g}}$ and $\sum _{g=1}^{f}{l}_{g}=l$. Each *D*_*g*_ is input separately to recover the seismic signals by system (11). Then all the sub-solutions *M*_*g*_, *g* = 1, 2, …, *f* are combined into *M* = [*M*_1_, *M*_2_, …, *M*_*f*_]. When the number of segments is two or three, the recovered err_rel_ values and SNR are almost the same as the integral case. When each column is considered as a segment, the joint matrix model is reduced to a sequence of vector recoveries, the recovery err_rel_ values and SNR are similar to the integral case but the computational time is around 50 times more.

### Reconstruction of seismograms from a heterogeneous model

**Figure 6:**
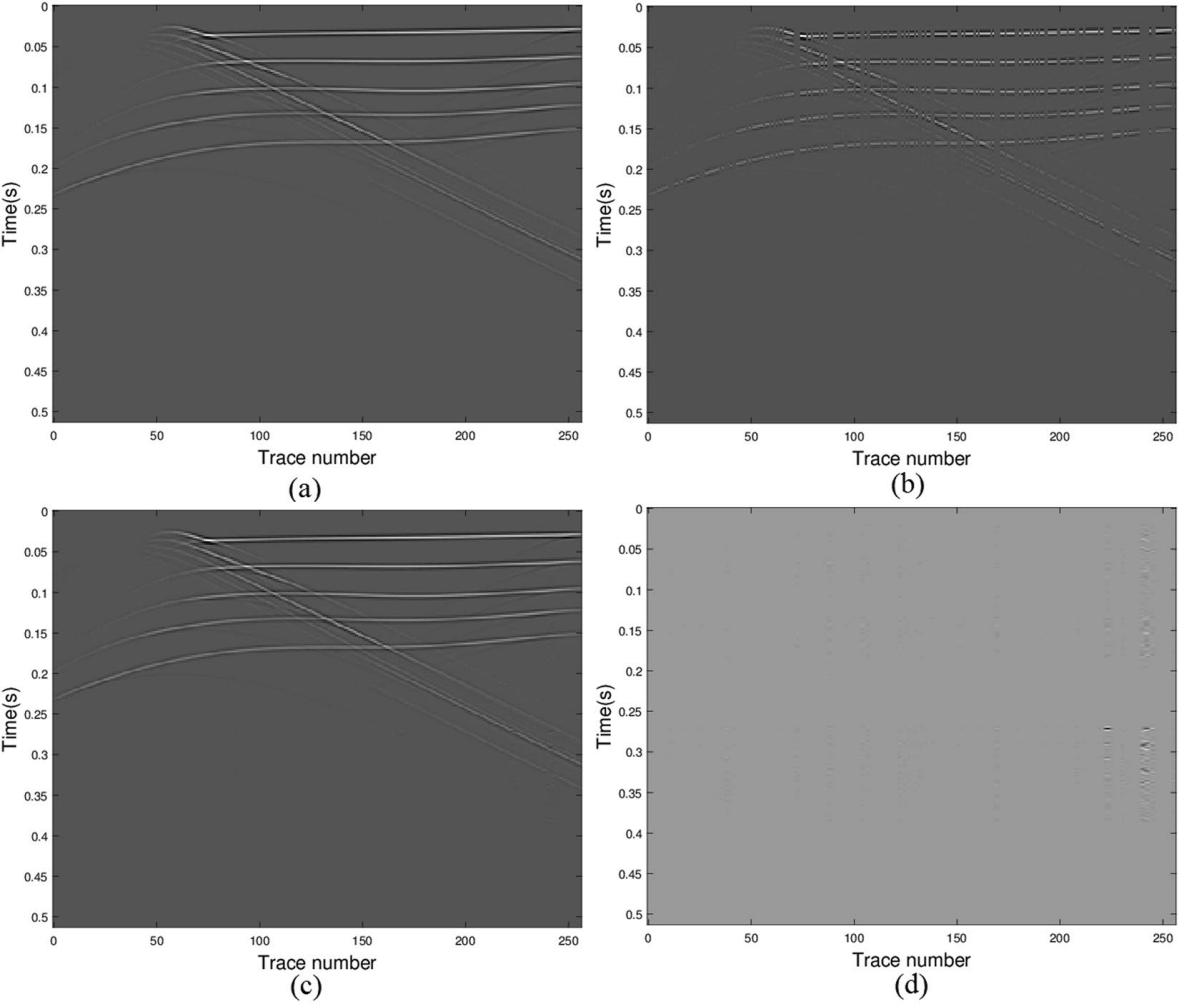
Seismic data results via model (11) with *q* = 2 and *p* = 0.5: (**a**) the real data; (**b**) the data with missing traces; (**c**) the recovered data; (**d**) error between the original and the recovered signals.

Next we consider a seismogram generated from a velocity model varying both vertically and transversely (Wang *et al*.^5^). The original seismic wavefield, sub-sampled data (37% traces are randomly removed) and recovered data are shown in Fig. 6(a–c), respectively. The difference of the original data and the recovered data is illustrated in Fig. 6(d). In displaying the results, the amplitude scale of the error map is the same as the amplitude scale of the data. It illustrates that all the initial seismic energy is recovered with minor errors. Though the reconstruction is not perfect, most of the details of the wavefield are preserved. Again, to test the quality of our algorithm in seismic data restoration for complex structure, we calculate the signal-to-noise ratio and the relative error. From our calculation, for *p* = 0.5, the values of SNR and err_rel_ are 26.9792 and 0.0448, respectively; for *p* = 1, the values of SNR and err_rel_ are 25.6940 and 0.0519, respectively. The high value of SNR and low value of err_rel_ indicate our algorithm works for seismic data restoration even with complex structure.

To show the robustness of our algorithm to interference, we add random noise with level 0.001 and 0.01 to the simulated data respectively. The unified Algorithm 3 with *p* = 0.5 is applied to solve the joint matrix minimization problems. The values of SNR and err_rel_ for noise level equaling 0.001 are 26.9074 and 0.0451, and for noise level equaling 0.001 are 18.0355 and 0.1254, respectively.

**Figure 7:**
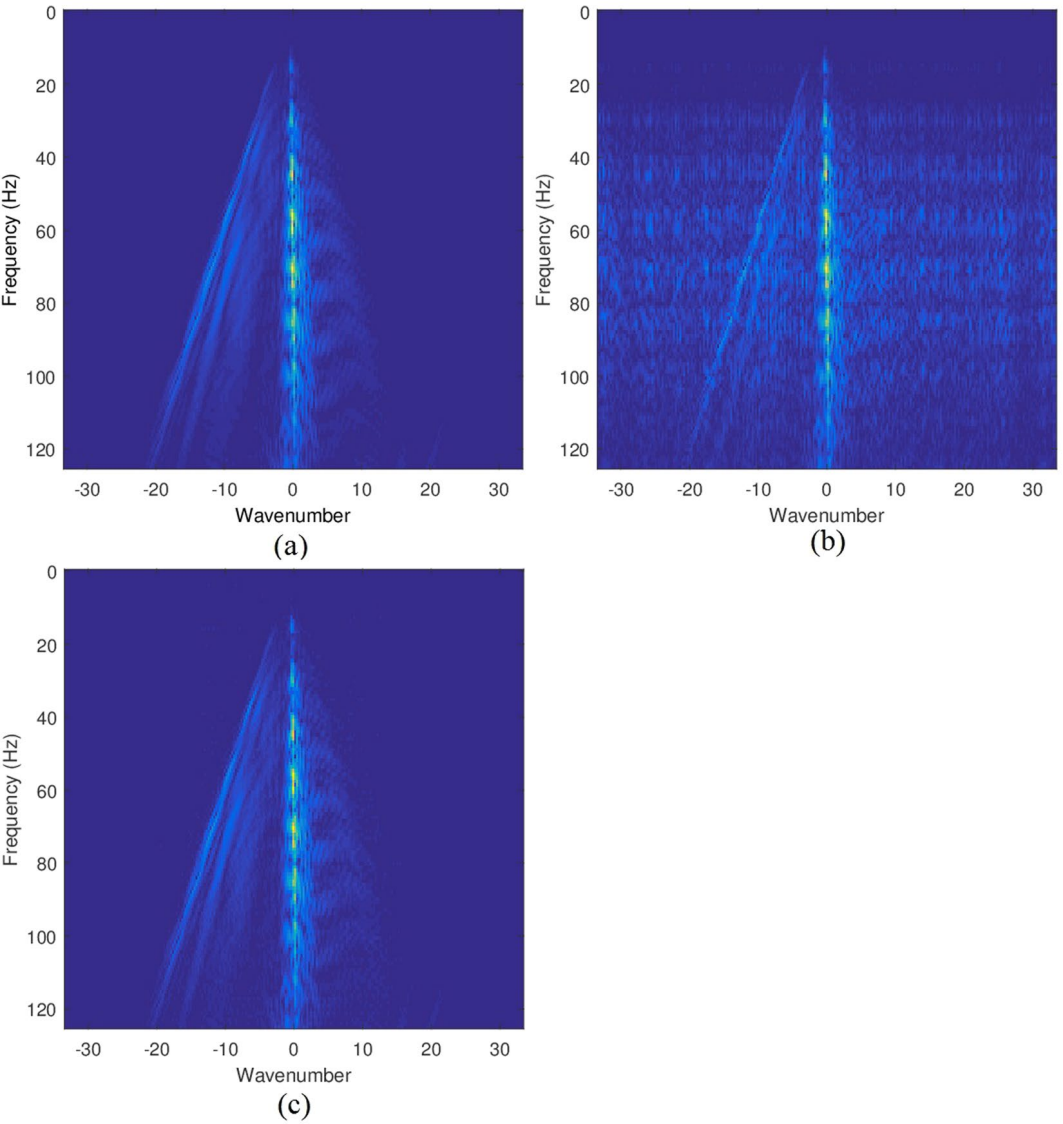
Frequency information: (**a**) frequency of the original data; (**b**) frequency of the sub-sampled data; (**c**) frequency of the restored data.

In the noisy case, e.g., noise level equaling 0.01, the frequency information of the original data, sub-sampled data and the recovered data are shown in Fig. 7(a–c), respectively. Again, the aliasing of the sub-sampled data is reduced greatly in the recovered data.

### Field data

**Figure 8:**
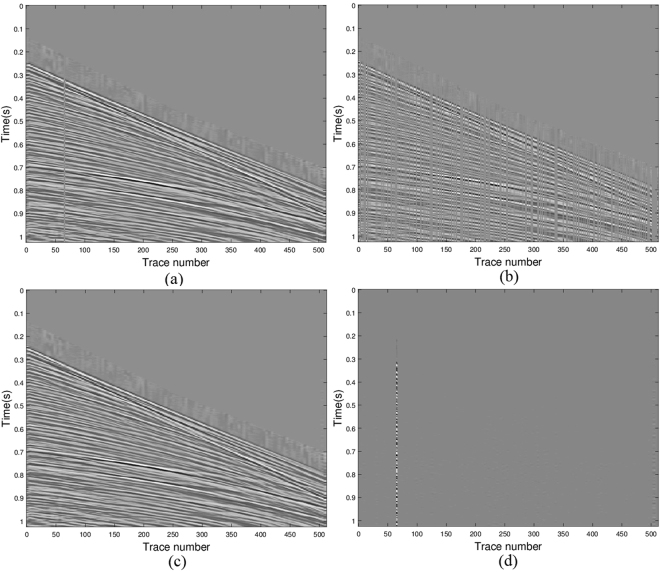
Seismic data results via model (11) with *q* = 2 and *p* = 0.5: (**a**) the real data, (**b**) the data with missing traces, (**c**) the recovered data and (**d**) error between the original and the recovered signals.

Finally, we examine the efficiency of the new method with field data. The seismic data is a marine shot gather shown in Fig. 8(a) which consists of 256 traces with spacing 25 m and time sampling interval 2 ms. There are damaged traces in the original gather. The subsampled gather is shown in Fig. 8(b) with 42% of the original traces randomly removed. This sub-sampled gather was used to restore the original gather with suitable solution methods. Again, the unified Algorithm 3 is applied to solve the joint matrix minimization problems (11) with *q* = 2 and *p* = 0.5. The recovery result is demonstrated in Fig. 8(c). The error of the original and the recovered data shown in Fig. 8(d) illustrates the efficient recovery performance of joint matrix minimization approach. In displaying the results, the amplitude scale of the error map is the same as the amplitude scale of the data. Comparing the subsampled image with the original image, the restored image can reconstruct most of the details. In addition the damaged trace in the original gather was restored as a good trace. Using the same definition of SNR as above, for *p* = 0.5, the value of SNR equals 19.7301; for *p* = 1 the value of SNR equals 19.7919. We only show figures for *p* = 1, since in visualization the results are similar for *p* = 0.5.

**Figure 9:**
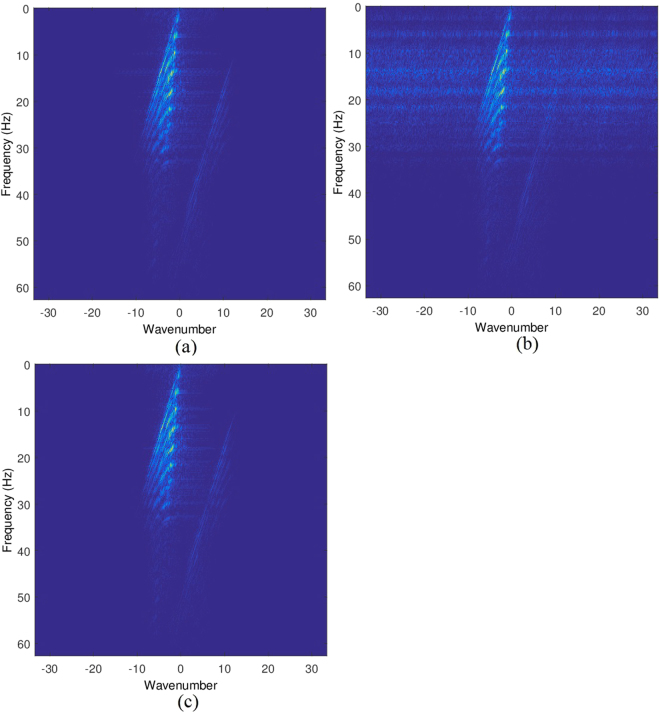
Frequency information: (**a**) frequency of the original data; (**b**) frequency of the sub-sampled data; (**c**) frequency of the restored data.

The frequency information of the original data, sub-sampled data and the recovered data are shown in Fig. 9(a–c), respectively. It indicates that the aliasing of the sub-sampled data is reduced greatly in the recovered data.

## Conclusion

Sparse optimization has broad applications in seismic data processing. In this paper we focus on data restoration problem. Noticing that the seismic wavefield can be represented using matrix instead of vector as weight variable to express all the signals simultaneously, in this paper we propose a matrix optimization model to the seismic wavefield recovery. We first reformulate the data restoration problem using an *l*_2, *p*_-norm constrained matrix minimization model for any *p* $\in$ (0, 1], which is a nonconvex and non-Lipschitz continuous minimization problem. Then we develop a unified algorithm to solve the mixed matrix optimization problem for any *p* $\in$ (0, 1]. Convergence analysis of the new algorithm is also addressed. Numerical results on synthetic problems and the field data example indicate potential usage of our method for practical applications.

### Appendix Properties of the new algorithms

In this section, we will analyze the convergence property of the Algorithm 2. The main result is that the objective function *J*(*M*_*t*_) strictly decreases with respect to iterations until the matrix sequence {*M*_*t*_} converges to a stationary point of *J*(*M*).

**Proposition 1**. Let $\phi (\tau )=\tau -a{\tau }^{\frac{1}{a}}$ be a function of the variable *τ*, where *a* $\in$ (0, 1). Then for any *τ* > 0, *φ*(*τ*) ≤ 1−*a*, and *τ* = 1 is the unique maximizer.

To verify the above statements, let us take the derivative of *φ*(*τ*) and set it to be zero, that is

$$\phi ^{\prime} (\tau )=1-{\tau }^{\frac{1}{a}-1}=0,$$

then *φ*′(*τ*) = 0 has the unique solution *τ* = 1 for any *a* $\in$ (0, 1) which is just the maximizer of *φ*(*τ*) in (0,  +∞).

Based on Proposition 1, for a given *a* $\in$ (0, 1),

A-1 $$\tau -a{\tau }^{\frac{1}{a}}\le 1-a$$

holds for *τ* $\in$ (0,  +∞) and “=’’ is active if and only if *τ* = 1. Let *a* takes special values such as $a=\frac{p}{2}\,(p\in (0,1])$, the inequality (A-1) will result in the following formula associated with $||M|{|}_{2,p}^{p}(0 < p\le 1)$.

**Proposition 2**. Suppose that *M*_*t*_ and ${\hat{M}}_{t+1}$ are generated in the *t*-th iteration by Algorithm 2, the following inequality holds,

A-2 $$\parallel {{\rm{\Lambda }}}_{t}{\hat{M}}_{t+1}{\parallel }_{2,p}^{p}-\frac{p}{2}\sum _{k\in {{\rm{\Omega }}}_{t}}\frac{{\alpha }_{k}\parallel {\hat{m}}_{t+1}^{k}{\parallel }_{2}^{2}}{\parallel {m}_{t}^{k}{\parallel }_{2}^{2-p}}\le (1-\frac{p}{2})\parallel {{\rm{\Lambda }}}_{t}{M}_{t}{\parallel }_{2,p}^{p},\,p\in (0,1],$$

where ${{\rm{\Lambda }}}_{t}=diag{\{{\alpha }_{k}\}}_{k\in {{\rm{\Omega }}}_{t}}$. Moreover, the equality in (A-2) holds if and only if $\parallel {\hat{m}}_{t+1}^{k}{\parallel }_{2}=\parallel {m}_{t}^{k}{\parallel }_{2}$ for *k* $\in$ Ω_*t*_.

Consider the approximate value *M*_*t*_. Since each ${\Vert {m}_{t}^{k}\Vert }_{2}\ne 0$ for *k* $\in$ Ω_*t*_, so we can r $\tau =\frac{{\Vert {\hat{m}}_{t+1}^{k}\Vert }_{2}^{p}}{{\Vert {m}_{t}^{k}\Vert }_{2}^{p}}$ and $a=\frac{p}{2}$ in Proposition 1. It can be obtained that

A-3 $$\frac{\parallel {\hat{m}}_{t+1}^{k}{\parallel }_{2}^{p}}{\parallel {m}_{t}^{k}{\parallel }_{2}^{p}}-\frac{p}{2}\frac{\parallel {\hat{m}}_{t+1}^{k}{\parallel }_{2}^{2}}{\parallel {m}_{t}^{k}{\parallel }_{2}^{2}}\le 1-\frac{p}{2},\quad k\in {{\rm{\Omega }}}_{t}\,.$$

Multiplying equation (A-3) by ${\alpha }_{k}\parallel {m}_{t}^{k}{\parallel }_{2}^{p}$, we have the following inequality

A-4 $${\alpha }_{k}{\Vert {\hat{m}}_{t+1}^{k}\Vert }_{2}^{p}-\frac{p}{2}\frac{{\alpha }_{k}{\Vert {\hat{m}}_{t+1}^{k}\Vert }_{2}^{2}}{{\Vert {m}_{t}^{k}\Vert }_{2}^{2-p}}\le (1-\frac{p}{2}){\alpha }_{k}{\Vert {m}_{t}^{k}\Vert }_{2}^{p},\quad k\in {{\rm{\Omega }}}_{t}.$$

Summing up *k* $\in$ Ω_*t*_ in formula (A-4}), we can derive at (A-2).

Based on Proposition 1, *τ* = 1 is the unique minimizers for *φ*(*τ*) in (0,  +∞) when $a=\frac{p}{2}$. Namely, ${\Vert {\hat{m}}_{t+1}^{k}\Vert }_{2}={\Vert {m}_{t}^{k}\Vert }_{2}(k\in {{\rm{\Omega }}}_{t})$ is necessary and sufficient for equality holding in (A-4). Now, we can establish the following convergence property of the Algorithm 2.

**Proposition 3**. Suppose that {*M*_*t*_} is the matrix sequence generated by Algorithm 2. Then *J*(*M*_*t*_) strictly decreases with respect to *t* for any 0 < *p* ≤ 1 until {*M*_*t*_} converges to a stationary point of *J*(*M*).

**Proposition 4**. Based on the derivation of Proposition 3, so long as the subproblem (16) is solved with ${Q}_{t}({\hat{M}}_{t+1})\le {Q}_{t}({M}_{t})$, the convergence of Algorithm 3 will be guaranteed for any *p* $\in$ (0, 1].
